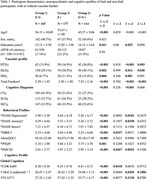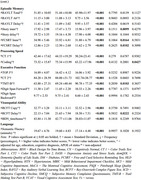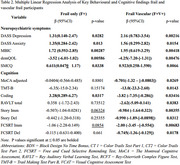# Frailty and Cerebrovascular Profiles Are Associated with Early Cognitive and Behavioral Impairment in a Community‐Based Southeast Asian Cohort

**DOI:** 10.1002/alz70857_102555

**Published:** 2025-12-24

**Authors:** Faith Phemie Hui En Lee, Yi Jin Leow, Pricilia Tanoto, Nagaendran Kandiah

**Affiliations:** ^1^ Dementia Research Centre (Singapore), Lee Kong Chian School of Medicine, Nanyang Technological University, Singapore 308232, Singapore, Singapore, Singapore; ^2^ nil, nil, nil, Nicaragua; ^3^ Dementia Research Centre (Singapore), Lee Kong Chian School of Medicine, Nanyang Technological University, Singapore, Singapore; ^4^ Neuroscience and Mental Health Programme, Lee Kong Chian School of Medicine, Nanyang Technological University, Singapore, Singapore; ^5^ Lee Kong Chian School of Medicine, Nanyang Technological University, Singapore, Singapore; ^6^ National Healthcare Group, Singapore, Singapore; ^7^ Duke‐NUS Medical School, National University of Singapore, Singapore, Singapore

## Abstract

**Background:**

Frailty and cerebrovascular disease (CSVD) frequently coexist, particularly in aging populations. In a meta‐analysis, frailty affected nearly half (49%) of stroke patients, with 22% classified as frail, contributing to poorer rehabilitation outcomes and greater disability. Despite growing evidence of their individual impacts, the synergistic effects of frailty and CSVD on cognitive and behavioral outcomes remain underexplored. With a rapidly aging global population, understanding these risk factors and outcomes is critical for developing targeted interventions. This study addresses this gap, and we hypothesize that frailty compounded by CSVD leads to more cognitive and behavioral impairments.

**Method:**

Cross‐sectional data from 738 participants (mean age: 57.49 ± 10.75; 40.7% male) BIOCIS study were analyzed. Participants were categorized into three groups: non‐frail, non‐CSVD (F‐V‐, *n* = 447); frail without CSVD (F+V‐, *n* = 177); and frail with CSVD (F+V+, *n* = 114). Frailty was assessed using the Fried Frailty Phenotype (pre‐frail: 1–2 points; frail: 3–5 points), while CSVD was graded using the modified Fazekas scale (absent‐to‐mild: 0–4; moderate‐to‐severe: 5–12).

**Result:**

Both frail groups (F+V‐ and F+V+) demonstrated significantly elevated neuropsychiatric symptoms (NPS) compared to F‐V‐, with the F+V+ group showing the most severe impairments. Depression (*p* = 0.01), higher Mild Behavioral Impairment Checklist (MBI‐C) scores (*p* = 0.003), poorer quality of life (demQOL, *p* <0.006), and increased subjective memory complaints (SMCQ, *p* = 0.03) were prominent among F+ participants. These impairments were exacerbated in F+V+, which demonstrated higher depression (*p* = 0.002) and anxiety (*p* = 0.015), and increased MBIC scores (*p* = 0.004). F+V+ group also reported poorer quality of life (demQOL, *p* < 0.005) and higher levels of SMCQ (*p* = 0.007).

Cognitive performance was similarly impaired in the F+V+ group, with deficits in executive function (TMT‐B, *p* = 0.01), processing speed (WAIS‐Coding, *p* = 0.03), and short‐term memory (RAVLT, *p* = 0.04; Story‐Immediate, *p* = 0.004; FCSRT‐Immediate, *p* = 0.007). Episodic memory impairments were evident, with lower scores in Story‐Delayed (*p* = 0.03) and FCSRT‐Delayed (*p* = 0.02).

**Conclusion:**

Frailty is primarily associated with increased NPS; however, the combination of frailty and CSVD amplifies cognitive and behavioral impairments. Our findings suggest CSVD exacerbates cognitive decline in frail individuals, creating a “double burden” of risk for accelerated deterioration. Early, integrated interventions targeting both frailty and cerebrovascular health are essential to mitigate this compounded risk and slow the trajectory of cognitive deterioration.